# Impact of pain characteristics and fear-avoidance beliefs on physical activity levels among older adults with chronic pain: a population-based, longitudinal study

**DOI:** 10.1186/s12877-016-0224-3

**Published:** 2016-02-24

**Authors:** Caroline Larsson, Eva Ekvall Hansson, Kristina Sundquist, Ulf Jakobsson

**Affiliations:** Center for Primary Health Care Research, Lund University/Region Skåne, Malmö, Sweden; Department of health Science, Lund University, Lund, Sweden; Stanford Prevention Research Center, Stanford University School of Medicine, Stanford, CA USA

**Keywords:** Older adults, Physical activity, Predictors, Kinesiophobia

## Abstract

**Background:**

To explore the level of physical activity in a population based sample of older adults; to analyze the influence of pain characteristics and fear-avoidance beliefs as predictors of physical activity among older adults reporting chronic pain.

**Methods:**

Demographics, pain characteristics (duration, intensity), physical activity, kinesiophobia (excessive fear of movement/(re) injury), self-efficacy and self-rated health were measured with questionnaires at baseline and 12-months later. Logistic regression analyses were done to identify associations at baseline and predictors of physical activity 12-months later during follow-up.

**Results:**

Of the 1141 older adults (mean age 74.4 range 65–103 years, 53.5 % women) included in the study, 31.1 % of those with chronic pain were sufficiently active (scoring ≥ 4 on Grimby’s physical activity scale) compared to 56.9 % of those without chronic pain. Lower age (OR = 0.93, 95 % CI = 0.88-0.99), low kinesiophobia OR = 0.95, 95 % CI = 0.91–0.99), and higher activity level at baseline (OR = 10.0, 95 % CI = 4.98–20.67) significantly predicted higher levels of physical activity in individuals with chronic pain.

**Conclusion:**

The level of physical activity was significantly lower among those with chronic pain and was significantly associated with kinesiophobia. Our findings suggest that fear- avoidance believes plays a more important role in predicting future physical activity levels than pain characteristics. Thus our findings are important to consider when aiming to increase physical activity in older adults that have chronic pain.

## Background

Chronic pain is common in older adults [1, 2] and is repeatedly found to be a significant risk factor for decreased functional capacity and onset and progression of disability in old age [1, 3, 4]. For example, studies of community-dwelling older adults have shown that pain is associated with falls [5], poorer physical performance [6], as well as elevated depression symptoms [7].

Physical activity is established as a significant moderator of both the physical and psychological dimensions of chronic pain, and hence an essential non-pharmacological strategy in the management of chronic pain [8, 9]. Moreover, physical activity is also known to decrease the risk of a myriad of health related problems such as type 2 diabetes, cardiovascular diseases and cancer as well as preventing disability, morbidity and mortality at old age [10–12]. Contrary to this, previous research has shown that older adults with chronic pain are less physically active when compared to older adults without chronic pain. For example, Griffin et al. (2012) found significantly lower levels of physical activity in older adults with chronic low back pain compared to asymptomatic individuals [13]. A recent meta-analysis by Stubbs et al. (2013) also showed that older adults with pain were significantly less active than those without chronic pain [14]. In general these studies were based on patients with back pain and the results suggested an increased risk for maintenance of pain but also for functional decline and additional chronic diseases, compared to older adults without chronic pain [11]. However, to increase the generalizability of the findings, the relationship between chronic pain and physical activity also needs to be investigated more generally, i.e., regardless of specific pain location in the body.

For healthy older adults the etiology of physical activity is well-known and includes risk factors such as: younger age, being male, being married, having better health scores, previous activity levels, self-efficacy, impaired mobility status and smoking [15–17]. Yet, there are reasons to believe that that the etiology of physical activity may differ in older adults that have chronic pain. Pain characteristics, such as duration of pain and pain intensity, have been identified as an important predictor for both self-reported and performance reported disability among older adults [4, 18] and may thus be assumed to interact with physical activity levels in older people with chronic pain. Another important consideration is fear-avoidance beliefs. The fear-avoidance model implies that daily activities and functional capacity may be reduced to avoid pain. Hence, untreated pain may lead to a negative spiral with the following: increased fear of movement, avoidance behavior and ultimately disuse, depression and further exacerbation of chronic pain [19, 20]. For the general population the existing literature is inconsistent regarding the relationship between fear-avoidance and physical activity levels. Some results suggest that fear-avoidance beliefs may not be directly associated with physical activity levels [13, 18, 21], but there are also several studies that conclude that the presence of fear-avoidance beliefs are significantly associated with increases in pain and reduced levels of physical activity [22, 23].

Despite the inconclusive results of previous research, most indications are that a relationship between the concepts can be assumed. However, the previous findings are primarily based on cross-sectional results. Thus the direct cause and influence of pain characteristics and fear avoidance beliefs on physical activity levels remains elusive. Identification of risk factors is important for prevention and can also allow identification possible targets for intervention. This is important when aiming to increase activity levels among older adults with chronic pain.

### Aim

The present study aimed to explore the level of physical activity in a population based sample of older adults, and to analyze the influence of pain characteristics and fear- avoidance beliefs as predictors of physical activity among older adults reporting chronic pain.

## Methods

### Participants

This longitudinal survey study included people aged 65 years and older, selected randomly using a Swedish national register of inhabitants (SPAR), which includes all persons who are registered as resident in Sweden. The randomization was performed by a government-engaged company (Infodata). All individuals 65 years or over were eligible for inclusion and no other exclusion criteria were used. Data were collected between May 2011 and May 2013. The sampling frame was Sweden’s whole population in the selected age group (65+ years). At baseline a total of 2,000 questionnaires were mailed out and 1,141 were completed and sent back. A total of 12 questionnaires were returned without having been filled in for the reason “address unknown” and 13 people were classified as deceased.

### Procedure and measurements

The baseline questionnaires were distributed by post together with an accompanying letter explaining the aim and procedure of the project. It was requested that the questionnaires be sent back with the enclosed self-addressed prepaid envelopes. Reminder letters were sent after a fortnight. For the 12-month follow-up we used the same procedure as for the baseline assessment, although no reminder letters were sent. The questionnaires contained demographic questions about: sex, age, BMI, smoking habits (“No, I have never smoked”, “Yes, but I have quit”, “Yes, occasionally”, and “Yes, daily”), housing (own home or special housing) and living arrangements (alone or with someone) and marital status.

Pain-related questions were also included in the questionnaires. Chronic pain, defined as pain that had lasted longer than three months [24] and was noted by responding YES or NO to the question “Have you been troubled by pain for the last three months or more?” Pain characteristic (intensity, duration, location) was measured using single items extracted from the brief Screening version of the Multidimensional Pain Inventory (Swedish version) [25]. Pain intensity was measured using the item “Rate the average level of your pain during the last week” with responses on a 6-point Likert scale ranging from *No pain at all* (1 point) to *Tremendous amount of pain* (6 points). Duration of pain was measured in years. Primary pain localization was also identified. The respondents could choose between 7 alternatives; upper extremities, shoulder and neck, lower extremities, thorax and abdomen, back and pelvis, head and other locations (including hand and feet). The brief Swedish version of MPI has been psychometrically tested and shown to have acceptable validity and reliability among the elderly, aged 60–89 years old [26].

*Kinesiophobia* (excessive fear of movement/(re) injury) related to pain was measured using an 11-item version of the *Tampa Scale of Kinesiophobia* (TSK-11) [27]. The 11 items each had four response options, all anchored with the answers *Strongly disagree*, which scored one point, and *Strongly agree*, which scored four points. A total summary score was then calculated and could range between 11 and 44 points. A high score indicated strong fear of movement/(re) injury, i.e. high kinesiophobia. The TSK-11 scale has been psychometrically evaluated and shown good construct validity and reliability in older adults (Cronbach’s alpha for internal consistency = 0.74–0.87) and good test-retest reliability (r = 0.747 for intraclass correlation (ICC)) [28].

*Self-efficacy* was measured using the *General Self-Efficacy scale* (GSE), a generic instrument that aims to measure “optimistic self-beliefs to cope with a variety of difficult demands in life”, recommended for use among adults with chronic pain [29]. The scale consists of 10 items with alternative responses: 1 = “not at all true”, 2 = “hardly true”, 3 = “moderately true”, and 4 = “exactly true”. A sum score, ranging from 10 to 40, is then calculated. A high score indicates high self-efficacy. The scale has commonly been used in older adults as well as in pain patients [30] and has been translated into Swedish [31]. The GSE has been tested for its psychometric properties and has demonstrated good validity and reliability (Cronbach’s alpha for internal consistency = 0.75–0.91) and good test-retest reliability (r = 0.55–0.67) [32].

Self-rated health was measured using an item extracted from the 12-item Short-Form Health Survey (SF-12) [33]. The SF-12 measures health-related quality of life. The item used in the present study was: “How would you generally like to say that your health is?” The item had five alternative responses: (“Excellent health”, “Very good health”, “Good health”, “Fair health” and “Poor health”). SF-12 has been found to be valid and reliable in Swedish older adults [34].

*Physical activity* recommendations from international health guidelines state that older adults should be moderately physically active (i.e. a moderate amount of effort that noticeably accelerates the heart rate) at least five days a week for a minimum of 30 min a day [35], but a study from 2011 suggested that only 30 % in the group aged 60+ years seemed to reach these recommended levels of physical activity [36]. Levels of physical activity were measured with *Grimby’s Activity Scale*; a scale developed to evaluate self-rated physical activity in older adults [37]. Levels of physical activity were classified using the question: “How physically active do you think you have been during the last six months?” Physical activity was classified by one of the following responses:

1. Hardly any physical activity.
2. Mostly sitting, sometimes a walk, light gardening or similar tasks, sometimes light household activities such as heating up food, dusting or clearing away.
3. Light physical exercise around 2–4 h a week, such as walks, fishing, dancing, ordinary gardening etc. including walks to and from shops. Main responsibility for light domestic work such as; cooking, dusting, clearing away and making beds. Performs or takes part in weekly cleaning.
4. Moderate exercise 1–2 h a week, such as jogging, swimming, gymnastics, heavy gardening, home-repairs or light physical activities more than four hours a week. Responsible for all domestic activities, light as well as heavy. Weekly cleaning such as doing vacuum cleaning, washing floors and window cleaning.
5. Moderate exercise at least three hours a week such as tennis, swimming, jogging etc.
6. Hard or very hard exercise regularly and several times a week where the physical exertion is great, such as jogging or skiing.

Response options 4–6 have previously been used to correspond to WHO’s recommended levels of physical activity [22]. The physical activity scale has been psychometrically evaluated in older adults and has demonstrated acceptable construct validity when validated against measures of physical performance [38, 39]. It also demonstrated acceptable construct validity when validated against various physical measures and has been found to be able to discriminate between groups, who were more active and less active, as assessed by measuring maximal oxygen uptake [38].

### Ethical considerations

The study was conducted in accordance with the basic ethical principles of medical research and was approved by the Regional Ethical Review Board in Lund (registration no. 2010/683). Written consent to participate was collected from all participants.

### Statistical analyses

Descriptive statistics are presented as the mean, standard deviation, and range, and percentiles were calculated. The Chi-squared test was used to compare categorical data; the Mann–Whitney U-test was used for ordinal data and the Student’s t-test for interval/ratio data. McNemar’s test, the Wilcoxon signed rank test, and the paired sample t-test were also used for paired group comparisons. To study the associations between the variables at baseline in those reporting chronic pain, two binary logistic regression analyses were done (by the backward stepwise likelihood ratio method) with physical activity, dichotomized as inactive (1–3) and active (4–6) [22], as the dependent variable. One logistic regression analysis was done to find associations at baseline and one logistic regression analyses was performed in order to find possible predictors of physical activity. Psychosocial and demographic variables were included based on empiric relations to physical activity found in previous studies [15–17, 20]. Demographic variables (housing and living arrangements), pain-related variables (intensity, duration), psychological variables (kinesiophobia and self-efficacy), and health-related variables (BMI, self-rated health) were entered as independent variables. To test the quality of the logistic models, the Hosmer–Lemeshow goodness-of-fit test and Nagelkerke’s R-squared were used. A value of *p* > 0.05 indicates a good model fit in the Hosmer–Lemeshow goodness-of-fit test [40]. Analyses were done using SPSS Statistics, version 18.0 (SPSS Inc., Chicago, IL).

## Results

This study included 1,141 people aged 65+ years (mean age 74.4 range 65–103 years, 53.5 % female). The response rate was 57.8 %. The only variable available for analysis for the non-responders (dropout analysis) was sex, for which no significant difference (*p* = 0.322) was found between participants (53.2 % women) and non-participants (55.8 % women).

Among the respondents, 433 (37.9 %) reported suffering from chronic pain for more than three months and they constituted the sample with chronic pain used for analyses in the present study (63.5 % women, mean age 74.8, 65–78 years). Of the 433 participants, who reported chronic pain in the baseline questionnaire, 284 responded to a follow-up questionnaire 12 months later. When comparing baseline characteristics in the chronic pain sample between those responding at baseline versus responding at follow up; only minor differences were found for: sex (63.5 % vs. 64.4 % women), pain intensity (3.2 vs. 3.2), duration (10.2 vs. 9.0), and kinesiophobia (22.8 vs. 21.9). However, regarding mean age (78.4 years vs. 74.39 years) the result indicated that those who were lost at follow-up were slightly older.

Characteristics for the sample at baseline are shown for chronic pain participants and non-chronic pain participants (Table 1). Significant differences between participants with chronic pain and those with no chronic pain were found for all variables, except for mean age and living condition (Table 1). In the total sample, 38.5 % of participants were found to be physically active. Assuming that none of the non-responders were physically active would give a lowest estimated prevalence of 33.3 %. However, assuming that all of the non-responders were active would give a total prevalence of 76.4 %. Participants with chronic pain were less active (31.1 %) than those without chronic pain (56.9 %) (*p* < 0.001). In the total population, men reported significantly higher levels of physical activity than women (*p* < 0.001), whereas among those suffering from chronic pain there was no gender difference (Table 1).

**Table 1:** Comparisons of demographic characteristics at baseline in participants with and without chronic pain^d^

Variable	Chronic pain (*n* = 433)	No chronic pain (*n* = 692)	*P*-value
Age, mean (SD) range	74.8 (7.5) 65-98	74.6 (7.0) 65-103	0.665^a^
Sex, n (%)			0.016^b^
Women	275 (63.5)	324 (46.8)	
Living conditions, n (%)			0.901^b^
Own accommodation	413 (97.4)	670 (97.5)	
Special housing	11 (2.6)	17 (2.5)	
Living arrangements, n (%)			0.037^b^
Alone	163 (38.3)	217 (32.1)	
With others	263 (61.7)	460 (67.9)	
Marital status, n (%)			0.038^c^
Married	235 (54.4)	414 (60.2)	
Single	38 (8.8)	58 (8.49)	
Widowed	95 (21.9)	137 (19.9)	
Divorced	64 (14.8)	79 (11.5)	
Physical activity^e^, n (%)			<0.001^c^
Hardly any physical activity	35 (8.4)	30 (4.5)	
Mostly sedentary	40 (9.6)	51 (7.7)	
Lighter physical exercise, around 2–4 h/week	211 (50.8)	295 (44.6)	
More strenuous exercise, 1–2 times/week	101 (24.3)	234 (35.4)	
More strenuous exercise, at least 3 h/week	23 (5.5)	31 (4.7)	
Hard regular training, several times/week	5 (1.2)	20 (3.0)	
Physical activity (dichotomized)^f^			<0.001^b^
Active	129 (31.1)	376 (56.9)	
Inactive	286 (68.9)	285 (43.1)	
Self-reported health, n (%)			<0.001^c^
Excellent	10 (2.3)	89 (13.0)	
Very good	61 (14.1)	194 (28.3)	
Good	146 (33.8)	276 (40.2)	
Fair	185 (42.8)	112 (16.3)	
Bad	30 (6.9)	15 (2.2)	
Self-efficacy^g^, mean (SD) range	30.1 (6.4) 10-40	31.5 (6.3) 10-40	<0.001^c^

^a^Student’s t-test

^b^Chi-square test

^c^Mann–Whitney U-test

^d^Pain of duration ≥3 months

^e^Assessed using Grimby’s activity scale

^f^Response options 1–3 as inactive and 4–6 as active

^g^Assessed using the General Self-Efficacy Scale (GSE). Scores range from 10 to 40 points with high scores indicating high self-efficacy

Among the participants with chronic pain, the mean duration of chronic pain was 10.2 (SD 12.2) years and the mean intensity 3.2 (SD1.1). Back/pelvis (34.1 %), lower extremities (30.7 %), followed by the upper extremities (13.4 %) were the three most common primary localizations of pain. Multiple locations were reported by 16.7 % of the participants. The mean level of kinesiophobia among those reporting chronic pain at baseline was 22.8 (SD 8.3). For the subsample with chronic pain, logistic regression analyses were performed to assess associations between baseline physical activity and variables previously shown to be related to physical activity levels. At baseline, low age, low BMI, high self-efficacy and lack of kinesiophobia showed statistically significant associations (*p* < 0.01) with physical activity (4–6 points on physical activity scale; Table 2).

**Table 2:** Associations with physical activity at baseline in older adults with chronic pain^a^

Variable	Odds ratio (OR)	95 % CI for OR		*P*-value
		Lower	Upper	
Age	0.889	0.844	0.936	<0.001
Sex	0.765	0.419	1.332	0.323
BMI	0.919	0.846	0.962	0.002
Self-efficacy	1.124	1.063	1.191	<0.001
Kinesiophobia	0.934	0.880	0.952	<0.001

^a^Pain of duration ≥3 months

The dependent variable, physical activity at baseline, was coded as 0 = inactive and 1 = active

The independent variables entered into the model were self-reported health (extracted from the SF-12), BMI, marital status, living conditions, smoking (yes/no), pain duration in years, pain intensity, self-efficacy (GSE) score, and kinesiophobia (TSK-11) score. The model was controlled for age (in years) and sex (0 = man, 1 = woman)

Nagelkerke’s R-square: 0.420

Hosmer–Lemeshow test: 0.819

For the subsample with chronic pain, a 12-month follow-up was used to identify potential predictors of physical activity levels. When comparing characteristics - in those reporting chronic pain - only the variables “Self-rated health” and “Pain intensity” showed significant differences between baseline and the 12-month follow-up (Table 3). For the variable “Pain intensity”, the score increased by 0.5 units and for the variable “Self-rated health” the response option “Poor health” showed the largest decrease in frequency, from 5.3 % at baseline down to 3.5 % at follow-up (Table 3).

**Table 3:** Characteristics of older adults with chronic pain^d^ who participated in both the baseline and 12-month follow-up assessments (*n* = 281)

Variable	Baseline	12 month follow-up	*P*-value
Physical activity^e^, n (%)			0.306^a^
Hardly any physical activity	15 (5.6)	13 (4.8)	
Mostly sedentary	21 (7.8)	23 (8.5)	
Lighter physical exercise, around 2–4 h/week	136 (50.4)	133 (49.3)	
More strenuous exercise, 1–2 times/week	69 (26.6)	82 (30.4)	
More strenuous exercise, at least 3 h/week	15 (5.8)	16 (5.9)	
Hard regular training, several times/week	3 (1.1)	3 (1.1)	
Physical activity^f^ (dichotomized), n (%)			0.194^b^
Active	87 (32.2)	101 (37.4)	
Inactive	172 (63.7)	169 (62.1)	
Self-reported health^g^, n (%)			0.046 ^a^
Excellent	7 (2.5)	7 (2.5)	
Very good	45 (16.0)	49 (17.4)	
Good	100 (35.5)	95 (33.7)	
Fair	114 (40.4)	121 (42.9)	
Bad	15 (5.3)	10 (3.5)	
Self-efficacy^h^, mean (SD) range	30.8 (6.2) 10–40	30.8 (6.4) 10–40	0.931^c^
Kinesiophobia^i^, mean (SD) range	22.2 (8.4) 11–44	22.7 (8.2) 11–44	0.972^c^
Pain duration^j^, mean (SD) range	10.0 (10.6) 0.5–60	10.0 (10.6) 0.1–50	0.554^c^
Pain intensity^k^, mean (SD) range	3.3 (1.0) 1–6	3.8 (1.0) 1–6	<0.001^c^

^a^Wilcoxon signed rank test

^b^McNemar’s test

^c^Paired t-test

^d^Pain of duration ≥3 months

^e^Assessed using Grimby’s activity scale

^f^Assessed using Grimby’s activity scale, response options 1–3 coded as inactive and 4–6 as active

^g^Assessed by a single item extracted from SF-12, "How would you generally like to say that your health is?"

^h^General self-efficacy scale (GSE) score, ranging from 10 to 40 points, with high scores indicating high self-efficacy

^i^Tampa Scale of Kinesiophobia (TSK-11) score, ranging from 11 to 44 points, with high scores indicating high kinesiophobia

^j^Pain duration in years

^k^Pain intensity = “average level of pain in the last week,” measured using a 6-point Likert scale, with answers ranging from *No pain at all* to *Tremendous amount of pain*

Table 4 shows the results of regression analysis focusing on potential predictors of physical activity at 12 months follow-up for the subsample with chronic pain. Younger age (OR = 0.9), baseline physical activity (OR = 10.0), and low scores on the kinesiophobia test (TSK-11) (OR = 0.9), all measured at baseline, predicted high levels of physical activity 12 months later.

**Table 4:** Predictors of physical activity at the 12-month follow-up in older adults with chronic pain^1^ (*n* = 216)

Variable	Odds ratio (OR)	95 % CI for OR		*P*-value
		Lower	Upper	
Age	0.930	0.875	0.989	0.020
Gender	0.746	0.367	1.516	0.418
Baseline physical activity	10.043	4.977	20.265	<0.001
Kinesiophobia	0.953	0.909	0.999	0.044

^1^ Pain of duration ≥3 months

The dependent variable, physical activity at 12 month follow up, was coded as 0 = inactive and 1 = active

The independent variables entered into the model were self-reported health (extracted from the SF-12), BMI, living conditions, smoking (yes/no), pain duration in years, pain intensity, self-efficacy (GSE) score, and kinesiophobia (TSK-11) score

The model was controlled for age (in years) and sex (0 = man, 1 = woman) and baseline physical activity (0 = inactive and 1 = active)

Nagelkerke’s R-square: 0.434

Hosmer–Lemeshow test: 0.206

## Discussion

The average level of physical activity was unchanged after 12-months. In the total population, men reported higher levels of physical activity (*p* < 0.001), whereas among those suffering from chronic pain there was no difference between the genders. Younger age, baseline physical activity and low kinesiophobia were associated with higher physical activity levels in older adults with chronic pain.

The prevalence of physical activity (4–6 on physical activity scale) differed significantly between older healthy adults (56.9 %) and older adults with chronic pain (31.1 %). Those in chronic pain reported significantly lower levels of physical activity compared to those without chronic pain (Table 1). A number of studies have illustrated a similar association for older adults with chronic low back pain [5, 13]. However, although the participants with chronic pain in this study were not substantially less active compared to activity levels previously reported for healthy older adults [36, 41, 42], the significantly lower levels of physical activity between the subgroups may suggest that older adults with chronic pain are at higher risk of functional decline and additional chronic diseases, compared to older adults without pain [11].

In the bivariate analysis, the variable “Pain intensity” showed a significant increase between baseline and the 12-month follow-up (Table 3). In the adjusted regression analyses, pain intensity was, however, not identified as a predictor of physical activity. Similar results have been found in studies including objective measurements of physical activity, indicating the impact of pain intensity to be more present in the establishment of pain behaviors than in its maintenance over time [18, 43]. The fact that pain intensity was not identified as a predictor of physical activity in our study can be explained by a strong correlation with other independent variables in the regression analysis. Then only one of them may be identified as a predictor. It is also notable that “Pain intensity” was registered as “average pain intensity during the last week” in our study. Pain intensity can thus, among these individuals (with, on average, a pain duration of 10 years), demonstrate temporal variation. It is also possible that the group of people that are characterized as “without pain” are actually people who don’t have chronic pain, but may have pain lasting less than 3 months.

Furthermore, the adjusted regression analyses indicate that the variables (age, baseline physical activity and kinesiophobia) rather than pain characteristics, such as pain-intensity and pain-duration, affect the level of physical activity in older adults with chronic pain. Age and baseline physical activity are well-known to be associated with physical activity levels in healthy older adults. Our findings, despite their conformity, are of major relevance because dissimilar factors might require diverse interventions in promoting physical activity in older adults with chronic pain, compared to those without. In addition it can be seen in this study that kinesiophobia relates to levels of physical activity, also when controlled for age, gender and baseline physical activity. The association to kinesiophobia in this study is in contrast to some previous studies conducted among general populations. In these studies it has been suggested that fear-avoidance beliefs are associated with disability through other mechanisms (such as type of activity), and not specifically with the amount or level of physical activity [13, 18, 21]. The relation of physical activity to the constructs of fear-avoidance among older adults in the current study might indicate that fear avoidance beliefs may be related directly to physical activity among older adults. This may be due to feelings of frailty and more severe consequences of a possible injury at old age. For example, a recent study identified avoidance of activities due to fear of falling as an important contributor of sedentary behavior among older adults with chronic muskoloskeletal pain [44]. Interventions targeted at reducing fear-avoidance beliefs (e.g. cognitive-behavioral therapies) have shown positive effects regarding both disability and the experience of pain [45]. Whether the same interventions also have an effect on the level of physical activity among older adults also needs further investigation. Nevertheless, the results indicate that fear avoidance-beliefs are important to consider in clinical settings and when designing treatment programs aiming to increase physical activity levels in older adults with chronic pain.

### Study limitations and strengths

This study has some limitations that need to be mentioned. Firstly, other factors than those investigated in this study, may underlie the association between chronic pain and physical activity in older adults. Such determinants may include comorbidity and socioeconomic variables but also factors relating to the built environment, such as walkability, may explain physical activity in older adults [46, 47]. Unfortunately these factors were not possible to consider in this study. Another possible confounder is cognitive status, which may have influenced both level and recall of physical activity. Unfortunately no screening for cognitive status was possible due to the study design, aiming to include population based sample of all older adults. Thus, the effects of cognitive status on physical activity and the role of proxy respondents have not been investigated. Secondly, the results of the study are based solely on self-reported measurements. Using more objective measures might have given slightly different results. For example, it must be considered that Grimby’s activity scale gives a quite crude measurement of physical activity. Objective measurement, using accelerometers for example, might have identified smaller changes and decreased the risk of recall bias. There is also documented under-estimation of inactivity for self-report questionnaires [48] and hence the true prevalence of inactivity may be even higher than that reported in the current study. However, a self-report questionnaire was deemed to be preferable due to the large sample size in this study. In addition, an important strength of Grimby’s activity scale is that it was developed for older adults and it has been shown to distinguish between active and non-active people. It is thus a superior choice for use in older adults compared to other available instruments for measuring physical activity, e.g., IPAQ, which has been shown to have questionable psychometric properties in this group [49].

The pain characteristics were measured using single items extracted from the brief Screening version of the Multidimensional Pain Inventory (Swedish version) [25]. Although the single items have not been psychometrically evaluated, the face validity is acceptable. We acknowledge that selection of these single items may complicate comparison with studies that used the full screening or different instruments.

Data were collected through questionnaires, which may have resulted in under-sampling of older adults who are not able to comprehend the data collection process (e.g., due to sight or cognitive difficulties). This may also have influenced the drop-out between baseline and follow-up and thus the oldest and possibly frailest elderly may not have been included. This means that the results may not reflect the attitudes and beliefs of the entire population of older adults with chronic pain. Systematic attrition and low response rates can be considered threats to internal validity. Generalization of our results must be done with this in mind. However, the response rate in the present study (57 %) is comparable to the rates for other studies of older adults [50]. Furthermore, an attrition analysis based on gender did not identify any differences when comparing our respondents to non-respondents sample, indicating that the drop-out may not be systematic regarding this variable.

## Conclusion

The level of physical activity was significantly lower among older adults with chronic pain compared to older adults without chronic pain. Low kinesiophobia was found to be associated with higher levels of physical activity among those with chronic pain, also when controlled for baseline physical activity and age. Our findings suggest that fear avoidance beliefs are important to consider, both in clinical settings and in research, when aiming to increase physical activity in older adults with chronic pain.

### Availability of data and materials

The dataset supporting the conclusions of this article is available on request. For further information on this database, you may contact the PI of the project, Ulf Jakobsson (ulf.jakobsson@med.lu.se).

## Abbreviations

SPAR: swedish personal address register
BMI: body-mass index
SPSS: statistical package for the Social Sciences
OR: odds ratio
TSK-11: tampa scale of kinesiophobia (11-item version)
GSE: general self-efficacy scale
SF-12: short form health survey
IPAQ: the international physical activity questionnaire

## References

[1] Patel KV, Guralnik JM, Dansie EJ, Turk DC (2013). Prevalence and impact of pain among older adults in the United States: findings from the 2011 national health and aging trends study. PAIN.

[2] Jakobsson U (2010). The epidemiology of chronic pain in a general population: results of a survey in southern Sweden. Scand J Rheumatol.

[3] Eggermont LHP, Leveille SG, Shi L, Kiely DK, Shmerling RH, Jones RN, Guralnik JM, Bean JF (2014). Pain characteristics associated with the onset of disability in older adults: the maintenance of balance, independent living, intellect, and zest in the elderly Boston study. J Am Geriatr Soc.

[4] Silva AG, Queirós A, Cerqueira M, Rocha NP (2014). Pain intensity is associated with both performance-based disability and self-reported disability in a sample of older adults attending primary health care centers. Disabil Health J.

[5] Stubbs B, Schofield P, Binnekade T, Patchay S, Sepehry A, Eggermont L (2014). Pain is associated with recurrent falls in community-dwelling older adults: evidence from a systematic review and meta-analysis. Pain Med.

[6] Pereira LSM, Sherrington C, Ferreira ML, Tiedemann A, Ferreira PH, Blyth FM, Close JCT, Taylor M, Lord SR (2014). Self-reported chronic pain is associated with physical performance in older people leaving aged care rehabilitation. Clin Interv Aging.

[7] Raftery MN, Sarma K, Murphy AW, De la Harpe D, Normand C, McGuire BE (2011). Chronic pain in the Republic of Ireland—community prevalence, psychosocial profile and predictors of pain-related disability: results from the prevalence, impact and cost of chronic pain (PRIME) study, part 1. PAIN.

[8] Bruce B, Fries J, Lubeck D (2005). Aerobic exercise and its impact on musculoskeletal pain in older adults: a 14 year prospective, longitudinal study. Arthritis Res Ther.

[9] Landmark T, Romundstad P, Borchgrevink PC, Kaasa S, Dale O (2011). Associations between recreational exercise and chronic pain in the general population: evidence from the HUNT 3 study. PAIN.

[10] Manini TM, Everhart JE, Patel KV (2006). Daily activity energy expenditure and mortality among older adults. JAMA.

[11] Sawatzky R, Liu-Ambrose T, Miller W, Carlo A. Physical activity as a mediator of the impact of chronic conditions on quality of life in older adults. In: Health Quality Life Outcomes. vol. 5; 2007. 10.1186/1477-7525-5-68 PMC2246116 18093310

[12] Tak E, Kuiper R, Chorus A, Hopman-Rock M (2013). Prevention of onset and progression of basic ADL disability by physical activity in community dwelling older adults: a meta-analysis. Ageing Res Rev.

[13] Griffin DW, Harmon DC, Kennedy NM (2012). Do patients with chronic low back pain have an altered level and/or pattern of physical activity compared to healthy individuals? a systematic review of the literature. Physiotherapy.

[14] Stubbs B, Binnekade TT, Soundy A, Schofield P, Huijnen IPJ, Eggermont LHP (2013). Are older adults with chronic musculoskeletal pain less active than older adults without pain? a systematic review and meta-analysis. Pain Med.

[15] Ashe MC, Miller WC, Eng JJ, Noreau L (2009). Older adults, chronic disease and leisure-time physical activity. Gerontology.

[16] Koeneman MA, Verheijden MW, Chinapaw MJ, Hopman-Rock M (2011). Determinants of physical activity and exercise in healthy older adults: a systematic review. Int J Behav Nutr Phys Act.

[17] Stralen MM, De Vries H, Mudde AN, Bolman C, Lechner L (2009). Determinants of initiation and maintenance of physical ativity among older people: a literature review. Health Psyhology Rev.

[18] Alschuler KN, Hoodin F, Murphy SL, Rice J, Geisser ME (2011). Factors contributing to physical activity in a chronic low back pain clinical sample: A comprehensive analysis using continuous ambulatory monitoring. PAIN.

[19] Vlaeyen JW, KoleSnijders AM, Rotteveel AM, Ruesink R, Heuts PH (1995). The role of fear of movement (re) injury in pain disability. J Occup Rehabil.

[20] Vlaeyen JW, Linton SJ (2000). Fear-avoidance and its consequences in chronic musculoskeletal pain: a state of the art. Pain.

[21] Heneweer H, Vanhees L, Picavet H, Susan J (2009). Physical activity and low back pain: A U-shaped relation?. PAIN.

[22] Elfving B, Andersson T, Grooten WJ (2007). Low levels of physical activity in back pain patients are associated with high levels of fear-avoidance beliefs and pain catastrophizing. Physiother Res Int.

[23] Nelson N, Churilla JR (2015). Physical activity, fear avoidance, and chronic non-specific pain: A narrative review. J Bodyw Mov Ther.

[24] International Association for the Study of Pain Subcommittee on Taxonomy. Classification of chronic pain. Descriptions of chronic pain syndromes and definitions of pain terms. Pain Suppl. 1986;3:S1-226. 3461421

[25] Jakobsson U, Horstmann V (2006). Psychometric evaluation of multidimensional pain inventory (Swedish version) in a sample of elderly people. Eur J Pain.

[26] Jakobsson U (2009). Psychometric testing of the brief screening version of multidimensional pain inventory (Swedish version). Scand J Caring Sci.

[27] Kori S, Miller R, Todd D (1990). Kineisiophobia: a new view of chronic pain behavior. Pain Manage.

[28] Larsson C, Hansson EE, Sundquist K, Jakobsson U (2014). Psychometric properties of the tampa scale of kinesiophobia (TSK-11) among older people with chronic pain. Physiother Theory Pract.

[29] Schwarzer R, Jerusalem M, Weinman J, Wright S, Johnston M (1995). Generalized self-efficacy scale. Measures in health psychology: a user’s portfolio causal and control beliefs.

[30] Luszczynska A, Scholz U, Schwarzer R (2005). The general self-efficacy scale: multicultural validation studies. J Psychol.

[31] Koskinen-Hagman M. Latent trait models of intrinsic, extrinsic, and quest religious orientations. *Doctorial* Lund: Lund University; 1999

[32] Scholz U, Doña BG, Sud S, Schwarzer R (2002). Is general self-efficacy a universal construct? psychometric findings from 25 countries. Eur J Psychol Assessment.

[33] Ware JEJ, Kosinski M, Keller SD (1996). A 12-item short-form health survey: construction of scales and preliminary tests of reliability and validity. Med Care.

[34] Jakobsson U (2007). Using the 12-item short Form health survey (SF-12) to measure quality of life among older people. Aging Clin Exp Res.

[35] WHO (2010). Global recommendations of on physical activity for health.

[36] Dumith SC, Hallal PC, Reis RS, Kohl Iii HW (2011). Worldwide prevalence of physical inactivity and its association with human development index in 76 countries. Prev Med.

[37] Saltin B, Grimby G (1968). Physiological analysis of middle-aged and old former athletes: comparison with still active athletes of the same ages. Circulation.

[38] Grimby G (1986). Physical activity and muscle training in the elderly. Acta Med Scand Suppl.

[39] Frändin K, Grimby G (1994). Assessment of physical activity, fitness and performance in 76-year-olds. Scand J Med Sci Sports.

[40] Hosmer DW, Lemeshow S (2000). Applied logistic regression.

[41] Hallal PC, Andersen LB, Bull FC, Guthold R, Haskell W, Ekelund U (2012). Global physical activity levels: surveillance progress, pitfalls, and prospects. The Lancet.

[42] Lim K, Taylor L (2005). Factors associated with physical activity among older people—a population-based study. Prev Med.

[43] Huijnen IPJ, Verbunt JA, Peters ML, Delespaul P, Kindermans HPJ, Roelofs J, Goossens M, Seelen HAM (2010). Do depression and pain intensity interfere with physical activity in daily life in patients with chronic low back pain?. PAIN.

[44] Stubbs B, Patchay S, Soundy A, Schofield P (2014). The Avoidance of activities due to fear of falling contributes to sedentary behavior among community-dwelling older adults with chronic musculoskeletal pain: a multisite observational study. Pain Med.

[45] Monticone M, Ferrante S, Teli M, Rocca B, Foti C, Lovi A, Brayda Bruno M (2014). Management of catastrophising and kinesiophobia improves rehabilitation after fusion for lumbar spondylolisthesis and stenosis. a randomised controlled trial. Eur Spine J.

[46] Van Cauwenberg J, De Bourdeaudhuij I, De Meester F, Van Dyck D, Salmon J, Clarys P, Deforche B (2011). Relationship between the physical environment and physical activity in older adults: A systematic review. Health Place.

[47] Yen IH, Michael YL, Perdue L (2009). Neighborhood environment in studies of health of older adults: a systematic review. Am J Prev Med.

[48] Harris TJ, Owen CG, Victor CR, Adams R, Cook DG (2009). What factors are associated with physical activity in older people, assessed objectively by accelerometry?. Br J Sports Med.

[49] Tomioka K, Iwamoto J, Saeki K, Okamoto N (2011). Reliability and validity of the international physical activity questionnaire (IPAQ) in elderly adults: the fujiwara-kyo study. J Epidemiol.

[50] Edelman LS, Yang R, Guymon M, Olson LM (2013). Survey methods and response rates among rural community dwelling older adults. Nurs Res.

